# Formation and spreading of TDP-43 aggregates in cultured neuronal and glial cells demonstrated by time-lapse imaging

**DOI:** 10.1371/journal.pone.0179375

**Published:** 2017-06-09

**Authors:** Tomohiro Ishii, Emiko Kawakami, Kentaro Endo, Hidemi Misawa, Kazuhiko Watabe

**Affiliations:** 1 Laboratory for Neurodegenerative Pathology, Tokyo Metropolitan Institute of Medical Science, Tokyo, Japan; 2 Division of Pharmacology, Faculty of Pharmacy, Keio University, Tokyo, Japan; 3 Center for Basic Technology Research, Tokyo Metropolitan Institute of Medical Science, Tokyo, Japan; 4 Department of Medical Technology (Neuropathology), Kyorin University Faculty of Health Sciences, Tokyo, Japan; "INSERM", FRANCE

## Abstract

TAR DNA-binding protein 43 (TDP-43) is a main constituent of cytoplasmic aggregates in neuronal and glial cells in cases of amyotrophic lateral sclerosis and frontotemporal lobar degeneration. We have previously demonstrated that adenovirus-transduced artificial TDP-43 cytoplasmic aggregates formation is enhanced by proteasome inhibition *in vitro* and *in vivo*. However, the relationship between cytoplasmic aggregate formation and cell death remains unclear. In the present study, rat neural stem cell lines stably transfected with EGFP- or Sirius-expression vectors under the control of tubulin beta III, glial fibrillary acidic protein, or 2′,3′-cyclic nucleotide 3′-phosphodiesterase promoter were differentiated into neurons, astrocytes, and oligodendrocytes, respectively, in the presence of retinoic acid. The differentiated cells were then transduced with adenoviruses expressing DsRed-tagged human wild type and C-terminal fragment TDP-43 under the condition of proteasome inhibition. Time-lapse imaging analyses revealed growing cytoplasmic aggregates in the transduced neuronal and glial cells, followed by collapse of the cell. The aggregates remained insoluble in culture media, consisted of sarkosyl-insoluble granular materials, and contained phosphorylated TDP-43. Moreover, the released aggregates were incorporated into neighboring neuronal cells, suggesting cell-to-cell spreading. The present study provides a novel tool for analyzing the detailed molecular mechanisms of TDP-43 proteinopathy *in vitro*.

## Introduction

Protein aggregation is one of the pathological hallmarks of neurodegenerative diseases such as prion disease, Alzheimer’s disease, frontotemporal lobar degeneration (FTLD), Huntington’s disease, Parkinson’s disease, and amyotrophic lateral sclerosis (ALS) [1–4]. TAR DNA-binding protein 43 (TDP-43) encoded by *TARDBP*, a DNA/RNA-binding protein that localizes predominantly to the nucleus and shuttles between the cytoplasm and nucleus [5], is detected as a main constituent of cytoplasmic aggregates in neuronal and glial cells in patients with ALS and FTLD with TDP-43-positive inclusions (FTLD-TDP) [6,7]. In pathological situations, TDP-43 is cleaved by the caspase and/or calpain family of proteases, which gives rise to a 25 kDa or a 35 kDa C-terminal fragment of TDP-43 (CTF TDP-43) localized to the cytoplasm [8]. Moreover, the CTF TDP-43 species are also generated by alternative start of translation [9]. These CTF TDP-43 contain conformationally unstable structures referred to as the prion-like domain and the glycine rich region and have aggregation-prone properties [10]. Interestingly, more than 40 mutations in *TARDBP* were reported in patients with ALS, most of which were localized in prion-like domain [4]. The CTF TDP-43 species with the ALS-linked mutations were reported to have more stable natures [11]. Cytoplasmic TDP-43 aggregates are likely composed of wild type (WT) and CTF TDP-43 [12], and have been shown to contain phosphorylated and ubiquitinated species of TDP-43 [13,14]. TDP-43 deposition is possibly mediated by multiple factors, such as impaired protein metabolism, stress granule formation, disrupted RNA metabolism, oxidative stress, neuroinflammation, and toxic factors released from astrocytes [15]. In addition, disrupted nuclear transport is also regarded as an important cause of aggregate formation especially that associated with C9orf72 [16,17]. Among these multiple factors involved in the TDP-43 pathogenesis, increasing lines of evidence support the notion that proteolytic pathways, including proteasome and autophagy systems, play key roles in TDP-43 aggregate formation [4,18–23]. Protein misfolding and aggregate deposition cause cellular dysfunction and ultimately lead to cell death [24,25]. We have previously demonstrated that proteasome inhibition enhanced the formation of adenovirus-transduced artificial TDP-43 cytoplasmic aggregates both *in vitro* and *in vivo*, suggesting that impairment of protein degradation pathways accelerates the formation of TDP-43-positive aggregates [26]. However, the molecular mechanisms underlying aggregate formation are largely unknown, and whether these aggregates play toxic or protective roles remains controversial [25,27].

Another prominent issue in current neurodegenerative disease research is elucidation of the mechanisms of disease progression in the central nervous system [28–31]. Prion-like propagation hypothesis is the one to explain the mechanism by which aggregates of disease-associated proteins are released and incorporated into adjacent cells; the incorporated aggregates act as seeds to form more aggregates [32,33].

In the present study, we performed time-lapse imaging analysis of neuronal and glial cells transduced with adenoviruses expressing TDP-43 to sequentially examine cytoplasmic aggregate formation, cell death, and cell-to-cell spreading of the aggregates.

## Results

### Cytoplasmic TDP-43 aggregate formation in rat neural stem cell-derived neurons and glial cells

**Fig 1:**
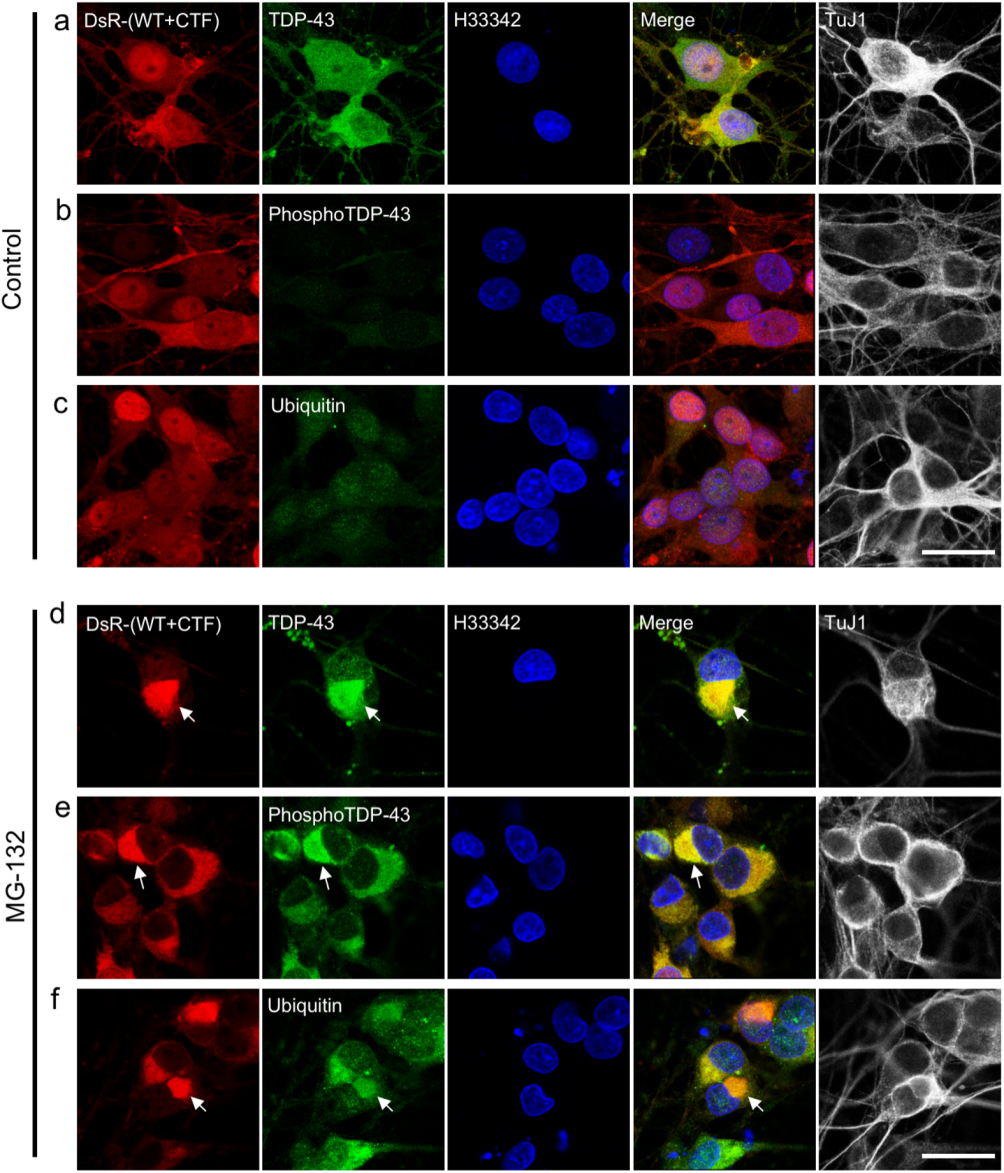
Formation of cytoplasmic TDP-43 aggregates is accelerated by proteasome inhibition in neuronally-differentiated 1464R cells. Differentiated 1464R neuronal cells were transduced with AxDsR-WT.TDP43 and AxDsR-CTF.TDP43 (DsR-(WT+CTF) (red) for 48 hrs and treated with DMSO (**a–c**) or MG-132 (**d–f**) for an additional 24 hrs. Fixed cells were immunostained for TuJ1 (**a–f**), TDP-43 (**a, d**), phosphorylated TDP-43 (pSer409/Ser410) (phosphoTDP-43) (**b, e**) and ubiquitin (**c, f**) with Alexa Fluor 488- or 633-conjugated secondary antibodies, and counterstained with Hoechst 33342 (H33342; blue). In MG-132-treated TuJ1-positive neurons (**a–c**), cytoplasmic aggregates (arrows) are strongly immunoreactive for TDP-43 (**d**), phosphoTDP-43 (**e**) and ubiquitin (**f**). Scale bar = 20 μm.

**Fig 2:**
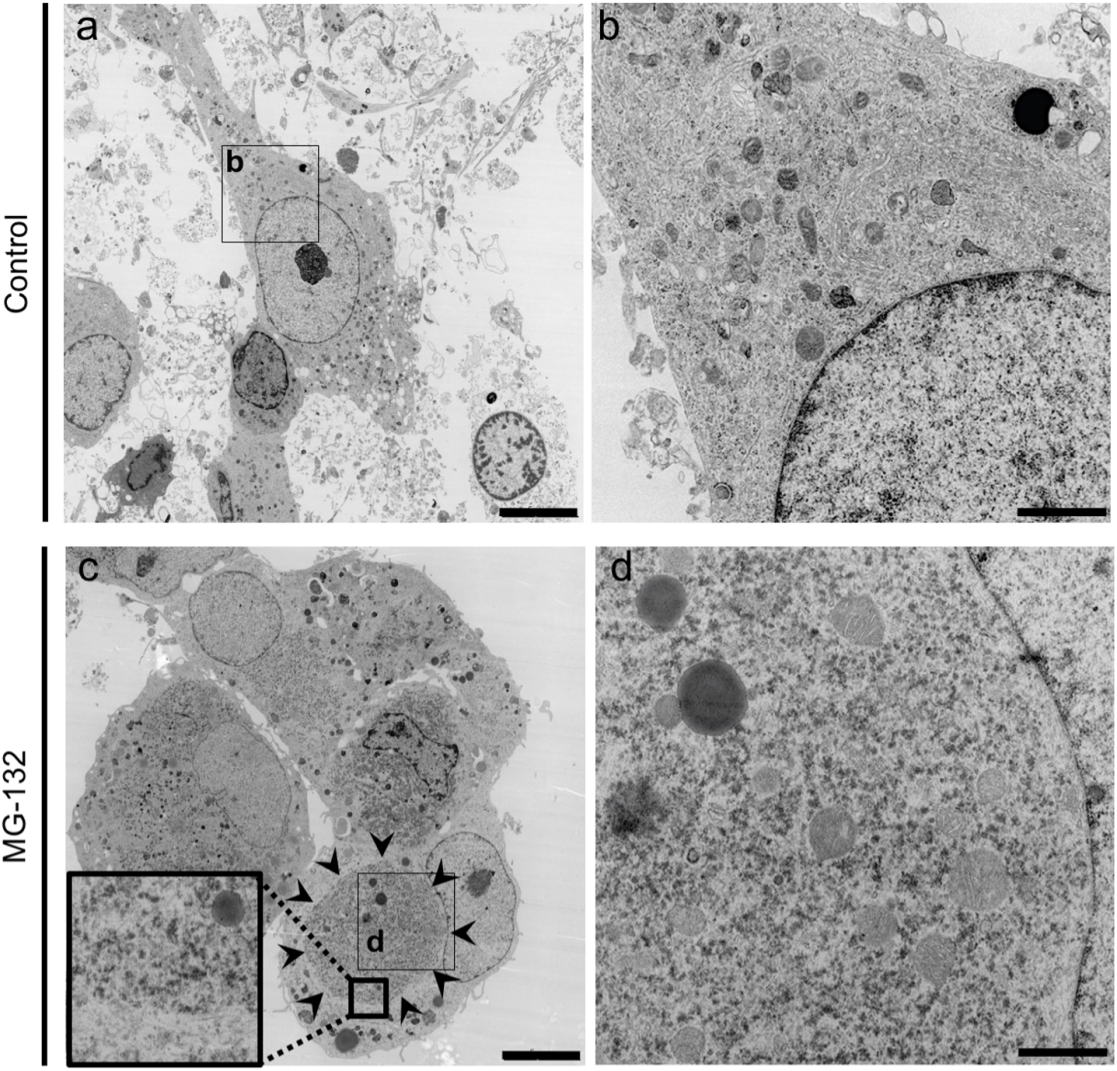
Cytoplasmic TDP-43 aggregates are composed of electron-dense granules. Electron micrographs of differentiated NSCs transduced with AxDsR-WT.TDP43 and AxDsR-CTF.TDP43 in the presence or absence of MG-132. (**a,b**) Cells transduced with AxDsR-WT.TDP43 and AxDsR-CTF.TDP43 are shown. (**c,d**) In cells transduced with AxDsR-WT.TDP43 and AxDsR-CTF.TDP43 followed by treatment of MG-132, non-membrane bound cytoplasmic aggregates (**c**; surrounded by arrowheads and magnified in inset) are composed of electron-dense fine granular materials intermingled with mitochondria and lysosomal vesicles (**d**). Scale bar = 5 μm (**a, c**), 1 μm (**b, d**).

In the present study we used a neural stem cell (NSC) line 1464R established from the adult rat brainstem [26] to examine aggregate formation of TDP-43. In the presence of retinoic acid, 1464R cells differentiate predominantly into TuJ1-positive neurons and, to a lesser extent, into GFAP-positive astrocytes or O4-positive oligodendrocytes. We have demonstrated cytoplasmic aggregate formation in these neurons and glial cells by co-transduction of adenoviruses expressing DsRed-tagged WT and CTF (208-414aa; 25kD) TDP-43 in the presence of proteasome inhibitor MG-132 [26]. In this study, we confirmed that these cytoplasmic TDP-43 aggregates were strongly immunoreactive for phosphorylated TDP-43 (Ser409/Ser410) and ubiquitin (Fig 1). The TDP-43 aggregate formation was more prominent when WT and CTF TDP-43 were co-transduced in the cells rather than when WT or CTF was transduced separately (Figs A-D in S1 Fig). Furthermore, similar aggregates were observed in the presence of MG-132 when DsRed tag was replaced with enhanced green fluorescent protein (EGFP), suggesting that the aggregate formation is not dependent on the types of fluorescent protein (Figs A-F in S2 Fig). Electron microscopically, these cytoplasmic aggregates were non-membrane bound, composed of electron-dense fine granular materials of 20–30 nm in diameter, and intermingled with mitochondria and lysosomal vesicles (Fig 2).

### Biochemical analysis of TDP-43 aggregates

**Fig 3:**
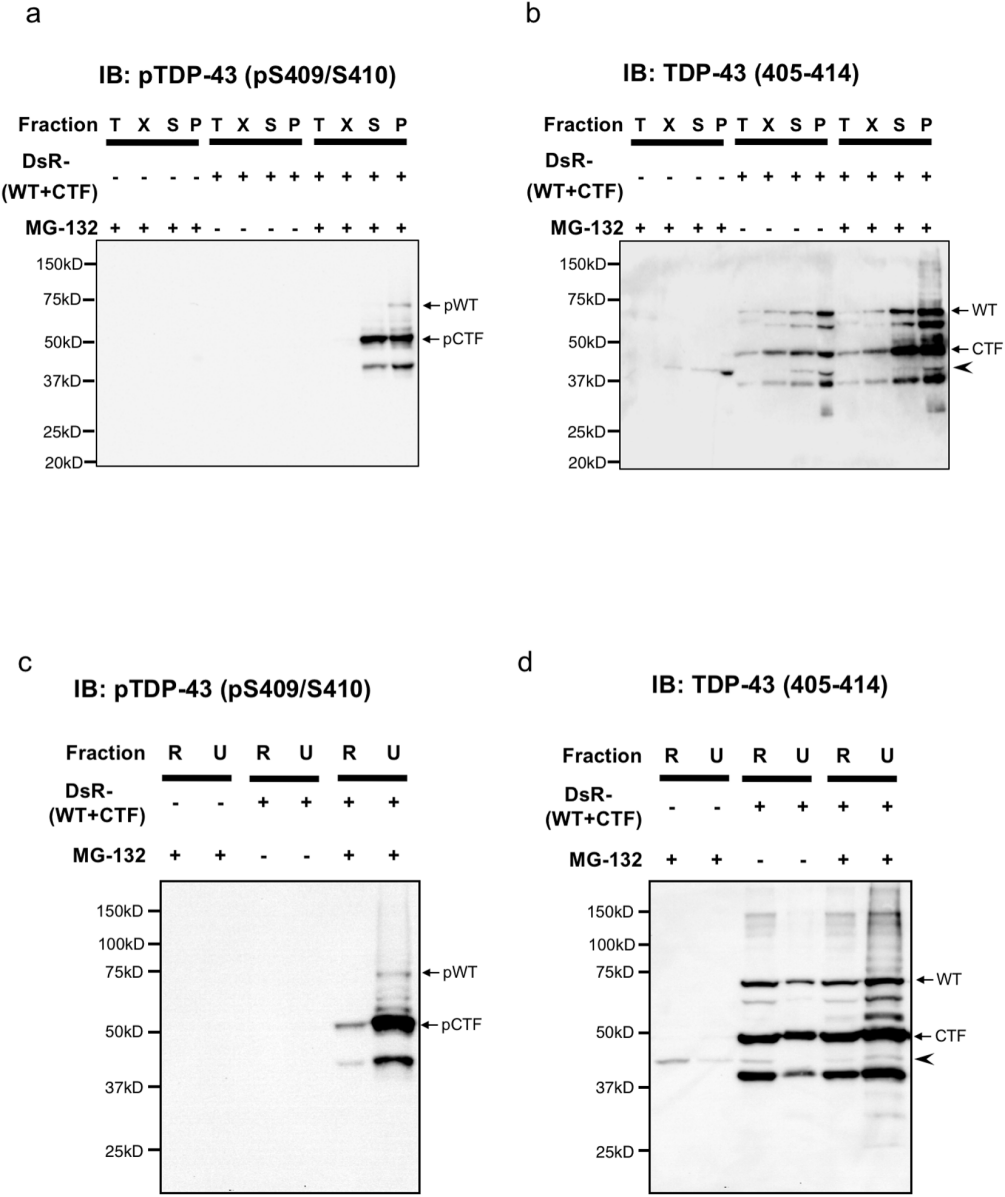
Adenovirus-transduced artificial TDP-43 cytoplasmic aggregates have insoluble properties against various detergents. (**a, b**) Sarkosyl-fractionated cell lysates with Tris (T), 1% TritonX-100 (X), 1% sarkosyl (S) and 1 × SDS-sample (P) buffers were examined using phospho (p)-TDP-43 (pSer409/Ser410) (**a**) or C-terminal TDP-43 (405–414) antibody (**b**). (**c, d**) RIPA/urea-fractionated cell lysates with RIPA (R) and urea (U) buffers were examined using pTDP-43 (**c**) or C-terminal TDP-43 (405–414) antibody (**d**). The 74 and 52 kDa bands correspond to phosphorylated DsRed-tagged WT (←pWT) and CTF (←pCTF) TDP-43, respectively (**a, c**). The 72 and 50 kDa bands correspond to non-phosphorylated DsRed-tagged WT (←WT) and CTF (←CTF) TDP-43, respectively. Endogenous rat TDP-43 band at 43 kDa was indicated as arrowhead (**b, d**). (**a-d**) Full length gels and blots are shown.

Since it has been widely established that pathological TDP-43 aggregates in ALS motor neurons are hyper-phosphorylated and ubiquitinated, which results in their resistance to detergent solubility, we next examined TDP-43 aggregates in our culture system biochemically by means of sarkosyl or RIPA/urea fractionation. In the case of sarkosyl fractionation followed by western blotting, phosphorylated DsRed-tagged WT and CTF TDP-43 signals were detected in MG-132-treated samples as a 74 kDa and a 52 kDa bands, respectively, both in the sarkosyl soluble and insoluble fractions (Fig 3A). In contrast, non-phosphorylated TDP-43 signals were observed in all fractions from TDP-43-transduced samples irrespective of MG-132 treatment, where 72 and 50 kDa bands corresponded to DsR-tagged WT and CTF TDP-43, respectively (Fig 3B). In the case of RIPA/urea fractionation followed by western blotting, phosphorylated DsRed-tagged WT and CTF TDP-43 bands were detected in both urea- and RIPA-soluble fractions from TDP-43-transduced and MG-132-treated samples (Fig 3C). Non-phosphorylated TDP-43 bands were observed in both RIPA and urea fractions from all TDP-43-transduced samples (Fig 3D). Consistent with a previous report by Hasegawa et al. [14], phosphorylated TDP-43 migrated slightly (approximately 2–3 kDa) behind non-phosphorylated TDP-43 in both sarkosyl and RIPA/urea fractionation analyses. Of note, phosphorylation predominantly occurred in CTF TDP-43 (Fig 3A and 3C), as was seen in previous reports [34] and in cases of FTLD-U and ALS patients [14], in spite of equimolar expression levels of WT and CTF TDP-43 proteins as examined by non-phosphorylated TDP-43 antibody (Fig 3B and 3D), suggesting the existence of yet unknown mechanisms involving selective phosphorylation of CTF TDP-43.

### Accelerated formation of TDP-43 aggregates and its phosphorylation by proteasome inhibition

To exclude the possibility that MG-132 has potential secondary effects on the other cell machineries besides proteasome, other proteasome inhibitors including lactacystin, epoxomicin and ALLN were examined. When DsRed-tagged WT and CTF TDP43-transduced 1464R cells were treated with lactacystin for 24 hrs, similar phosphorylated cytoplasmic aggregates were detected (Figs A-C in S3 Fig). Moreover, sarkosyl soluble and sarkosyl insoluble lysates from the cells treated with lacacystin, epoxomicin or ALLN showed similar biochemical properties as was observed in MG-132-treated cells (compare Fig 3, Figs D and E in S3 Fig). These results strongly suggest that proteasome inhibition enhances the formation of TDP-43 aggregates.

### Time-lapse imaging of cytoplasmic TDP-43 aggregate formation and cell death

**Fig 4:**
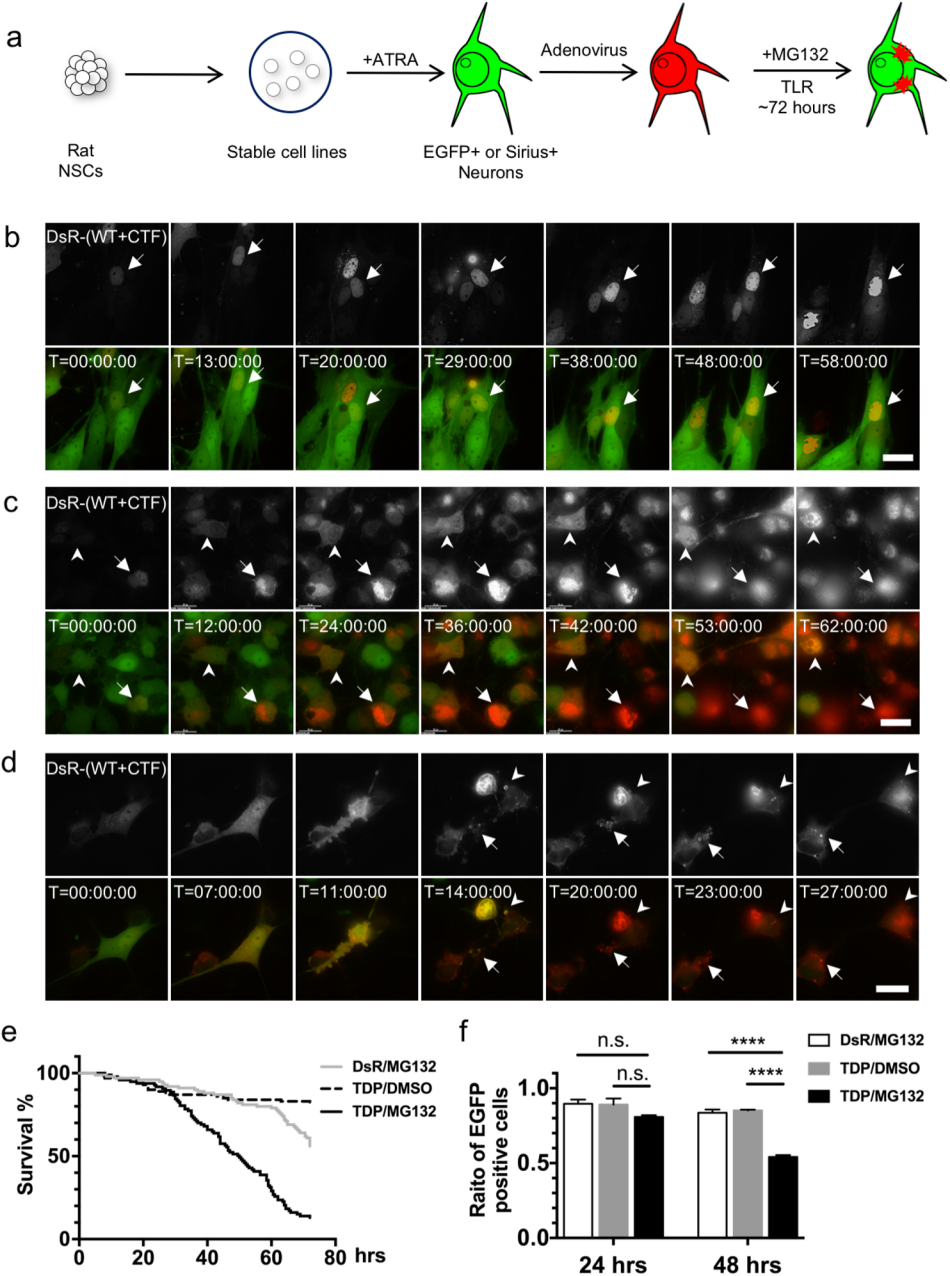
Formation of TDP-43 aggregates and neuronal cell death demonstrated by time-lapse imaging. (**a**) Schematic drawing illustrating the time-lapse imaging experiments. (**b–d**) Time-lapse imaging of AxDsR-WT.TDP43 and AxDsR-CTF.TDP43 (DsR-(WT+CTF); gray scale in top panels and red in bottom panels)- transduced 1464RTBB3pEGFP-derived neurons (green) in the absence (**b**) or presence (**c, d**) of MG-132. (**c**) DsRed-positive cytoplasmic aggregates are formed about 12 hrs after MG-132 treatment. The EGFP-positive cell bodies (arrows and arrowheads) are gradually swollen and finally lead to collapse within 72 hrs. These DsRed-positive aggregates remain insoluble in culture media for over 30 hrs of the time course. (**d**) DsRed-positive extracellular TDP-43 aggregates burst out of the cells and are taken up by neighboring EGFP-positive neurons (arrows and arrowheads). (e) Kaplan-Meier survival curve of DsR/MG132 group (n = 100), TDP/DMSO group (n = 100) and TDP/MG132 group (n = 97). (f) EGFP-positive cells in each group were counted at 24 hrs and 48 hrs after DMSO or MG-132 treatment. The number of cells in each experiment at 0 hrs was set as ratio 1.0 and the cell ratio was calculated at each point. Ratios of three independent experiments were graphed with mean ± SEM and analyzed by two-way ANOVA with Sidak’s multiple comparisons test. n.s., not statistically significant, **** p<0.0001.

To analyze TDP-43 aggregate formation and resulting cell alterations, we performed time-lapse imaging analysis. To visualize TDP-43 protein in living neurons by fluorescence microscopy, we established in the present study stable cell lines that express EGFP and Sirius (one of the blue fluorescent proteins) under the control of tubulin beta III (TBB3) promoter, designated 1464RTBB3pEGFP and 1464RTBB3pSirius respectively, from 1464R neural stem cells [26]. These cells were differentiated into EGFP (Fig 4B–4D)- or Sirius (Fig 4E–4G)-positive neurons in the presence of retinoic acid, transduced with adenoviruses expressing DsRed-tagged WT and CTF TDP-43 in the absence or presence of MG-132, and subsequently analyzed by time-lapse imaging for 72 hrs (Fig 4A). Most of the TDP-43-transduced cells without treatment of MG-132 were viable by the end of imaging analysis, showing DsRed fluorescence diffusely localized in their nuclei and cytoplasm, although very few cells were seen collapsing to death probably due to much higher expression of nuclear adenoviral DsRed-tagged TDP-43 (Fig 4B and S1 Movie). In the presence of MG-132, DsRed-positive cytoplasmic granules emerged, gradually increasing in amount and forming aggregates (Fig 4C and S2 Movie). As the DsRed-positive cytoplasmic aggregates grew larger, nuclear DsRed fluorescence became disappeared, suggesting cytoplasmic translocation of transduced full length TDP-43. The cell bodies were gradually swollen and finally collapsed within 72 hrs as the EGFP fluorescence dispersed out of the cytoplasm. These DsRed-positive aggregates remained visible as insoluble materials in the culture media for over 30 hrs of the time course (Fig 4C and S2 Movie). In some instances, the DsRed-positive extracellular TDP-43 aggregates burst out of the cells and were taken up by neighboring EGFP-positive neurons (Fig 4D and S3 Movie), suggesting cell-to-cell spreading of TDP-43 aggregates.

Next, quantitative analyses for cell survival were performed by the time-lapse imaging. The 1464RTBB3pEGFP cells were transduced with DsRed (as a control) followed by MG-132 (DsR/MG132), or WT and CTF TDP-43 followed by DMSO (TDP/DMSO) or MG-132 (TDP/MG132). Kaplan-Meier analysis showed ratio of cell survival (EGFP-positive cells containing aggregates were counted) was statistically different between DsR/MG132 and TDP/MG132 groups (p<0.0001) and between TDP/DMSO and TDP/ MG132 groups (p<0.0001) (Fig 4E). Although the ratio of EGFP-positive neurons were not significantly changed at 24 hrs between three groups (DsR/MG132 vs TDP/MG132; p = 0.110, TDP/DMSO vs TDP/MG132; p = 0.145), TDP/MG132 group showed significant decrease in the ratio of EGFP-positivity at 48 hrs compared to DsR/MG32 group or TDP/DMSO group (DsR/MG132 vs TDP/MG132; p<0.0001, TDP/DMSO vs TDP/MG132; p<0.0001) (Fig 4F).

**Fig 5:**
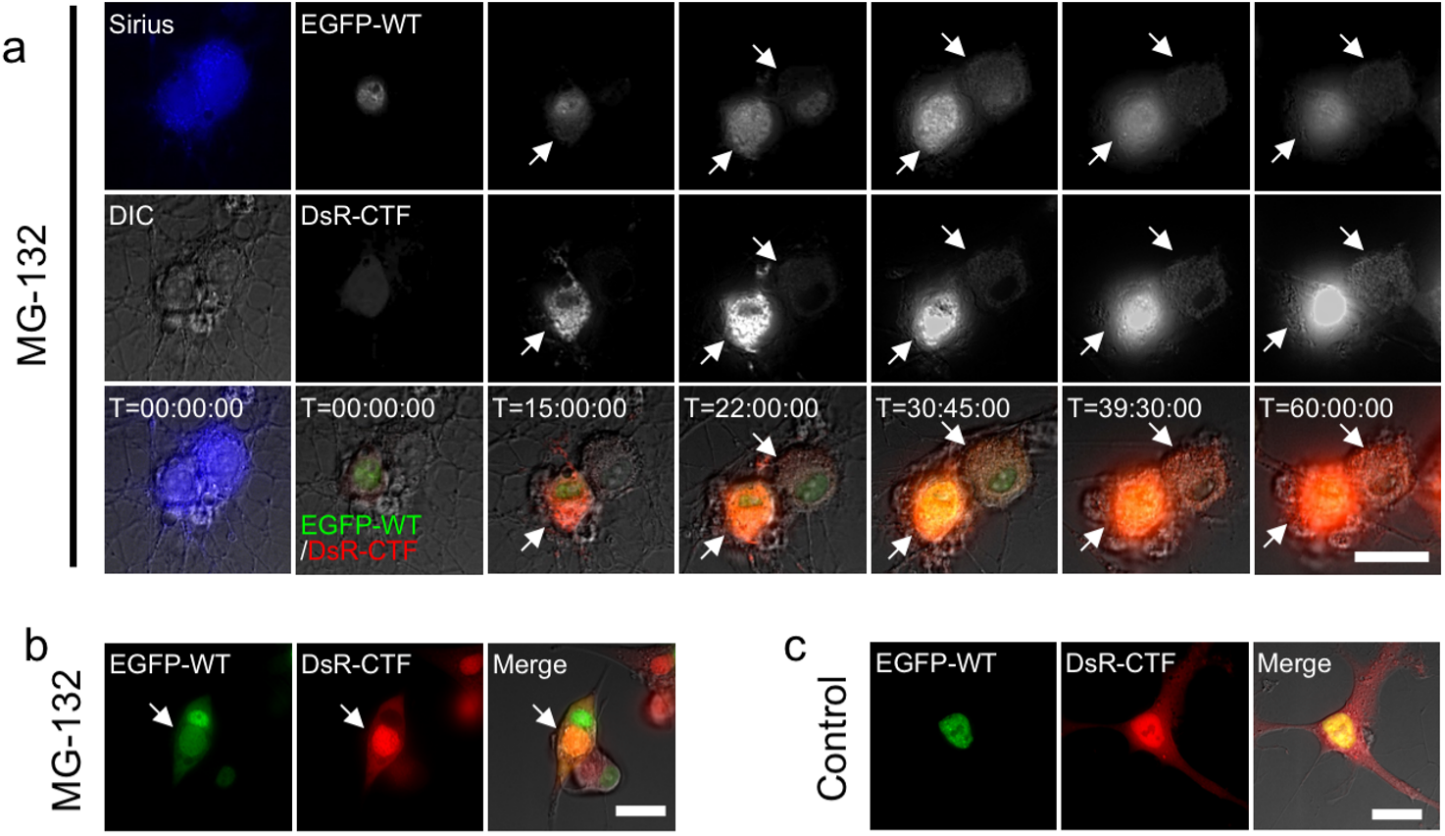
Cytoplasmic TDP-43 aggregates are composed predominantly of CTF. (**a**) Time-lapse imaging of AxEGFP-WT.TDP43 and AxDsR-CTF.TDP43-transduced 1464RTBB3pSirius neurons in the presence of MG-132 (EGFP-WT; gray scale in the top panel and green in the bottom panel, DsR-CTF; gray scale in the middle panel and red in the bottom panel). Cytoplasmic TDP-43 aggregates show both EGFP and DsRed fluorescence (arrows). (**b,c**) Snapshots showing live cells at the end of the time-lapse imaging in the presence (**b**) or absence (**c**) of MG-132. (**b**) In the presence of MG-132, cytoplasmic aggregates are composed of WT TDP-43 (EGFP-WT) (green) and CTF TDP-43 (DsR-CTF) (red) (arrows). Scale bar = 20 μm. See also S4 Movie.

When Sirius-positive neurons differentiated from 1464RTBB3pSirius cells (with blue fluorescence) were transduced with adenoviruses expressing EGFP-tagged WT and DsRed-tagged CTF TDP-43 in the presence of MG-132, cytoplasmic TDP-43 aggregates showed both EGFP and DsRed fluorescence, indicating that these aggregates consisted of both WT and CTF TDP-43 (Fig 5A and S4 Movie). Following cell collapse, released aggregates floating in culture media became EGFP-weakly positive and DsRed-positive, suggesting that CTF TDP-43 in aggregates gained more insoluble nature as compared to WT TDP-43. To exclude the possibility that DsRed protein has more insoluble nature compared with EGFP, EGFP-tagged WT and CTF TDP-43 were transduced with 1464RTBB3pSirius cells and time-lapse experiments were performed after MG-132 treatment. EGFP-positive cytoplasmic aggregates were gradually formed, these cells were collapsed with plasma membrane rapture, and EGFP-positive aggregates were still visible after cell death (Fig A in S4 Fig and S5 Movie). Furthermore, 1464RTBB3pSirius cells were co-transduced with DsRed-tagged WT TDP-43 and EGFP-tagged CTF TDP-43, and time-lapse imaging was performed in the presence of MG-132. As shown in Fig B in S4 Fig, cytoplasmic TDP-43 aggregates were formed by EGFP-tagged CTF TDP-43 and to a lesser extent by DsRed-tagged WT TDP-43. After cell death, insoluble TDP-43 aggregates were DsRed-weakly positive and EGFP-positive (Fig B in S4 Fig and S6 Movie), again suggesting more insoluble nature of CTF TDP-43 as compared to WT TDP-43.

**Fig 6:**
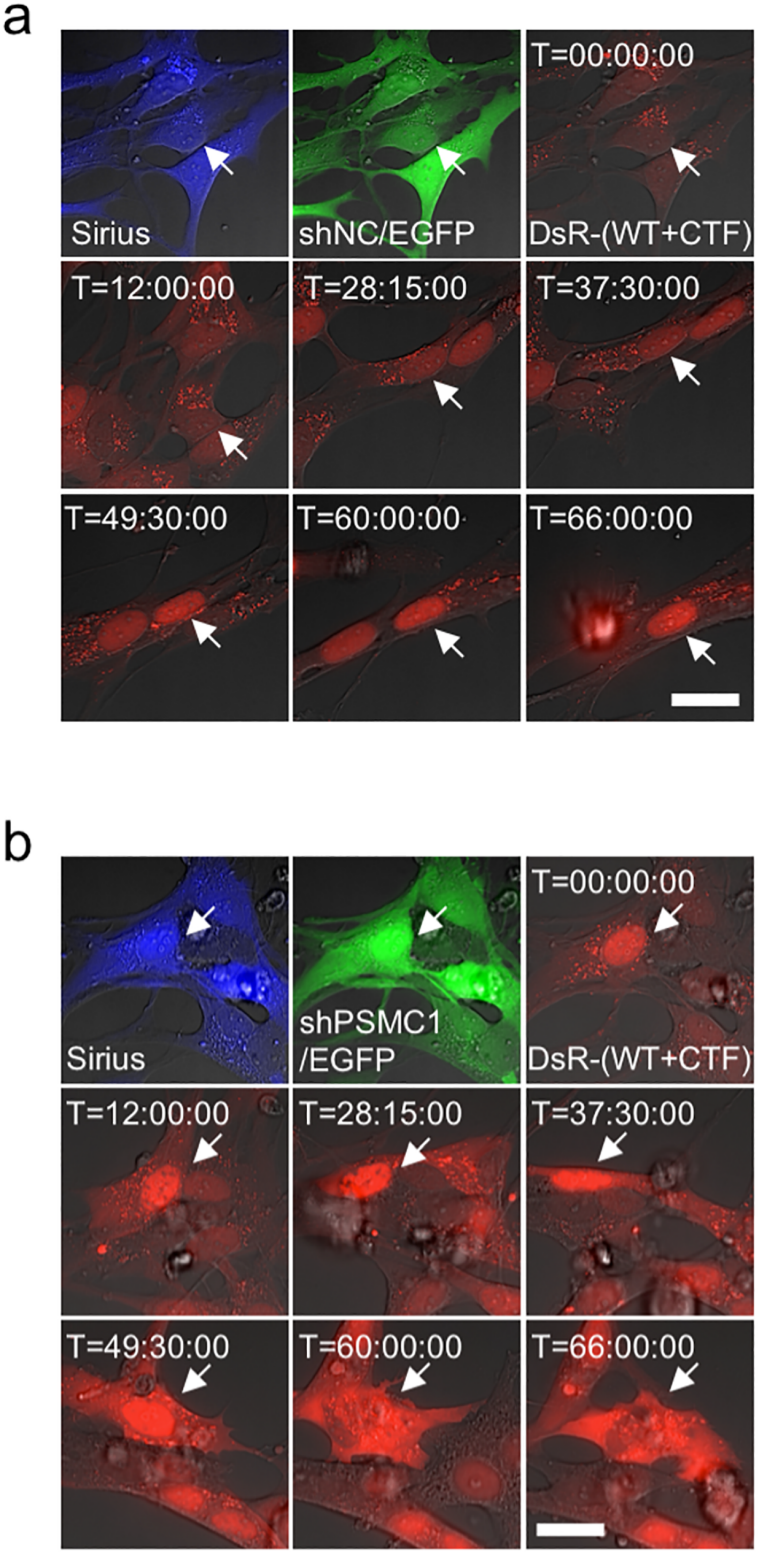
Inhibition of proteasome using shRNA for PSMC1 exhibits translocation of TDP-43. Time-lapse imaging of aggregate formation following translocation of transduced TDP-43 by expressing shRNA for proteasome subunit PSMC1 in 1464RTBB3pSirius neurons. Sirius-positive neurons transduced with AxDsR-WT.TDP43 and AxDsR-CTF.TDP43 along with AxshNC/EGFP (negative control) (a) or AxshPSMC1/EGFP (b) are shown. Proteasome inhibition by shPSMC1 induces cytoplasmic aggregate formation and translocation of TDP-43 (**b**; middle and bottom panels). Sirius (blue), EGFP (green) and DsRed-TDP-43 (DsR-(WT+CTF)) (red) signals are initially captured (top panels) and DsRed-TDP-43 signals are traced for 72 hrs (middle and bottom panels). Scale bar = 20 μm. Arrows show the position of same cell for tracking purpose.

Since previous study showed that cytoplasmic TDP-43 aggregates were formed by suppressing PSMC1, one of the key components in the proteasome complex, in adult rat and mouse motor neurons [26], Sirius-positive neurons differentiated from 1464RTBB3pSirius cells were transduced with adenoviruses expressing DsRed-tagged WT and CTF TDP-43 as well as shRNA for PSMC1 (shPSMC1) coupled with EGFP, similar results were obtained as those with the aforementioned MG-132 treatment, indicating that the inhibitory effect of adenoviral shPSMC1 is comparable to that of MG-132 (Fig 6A and 6B).

**Fig 7:**
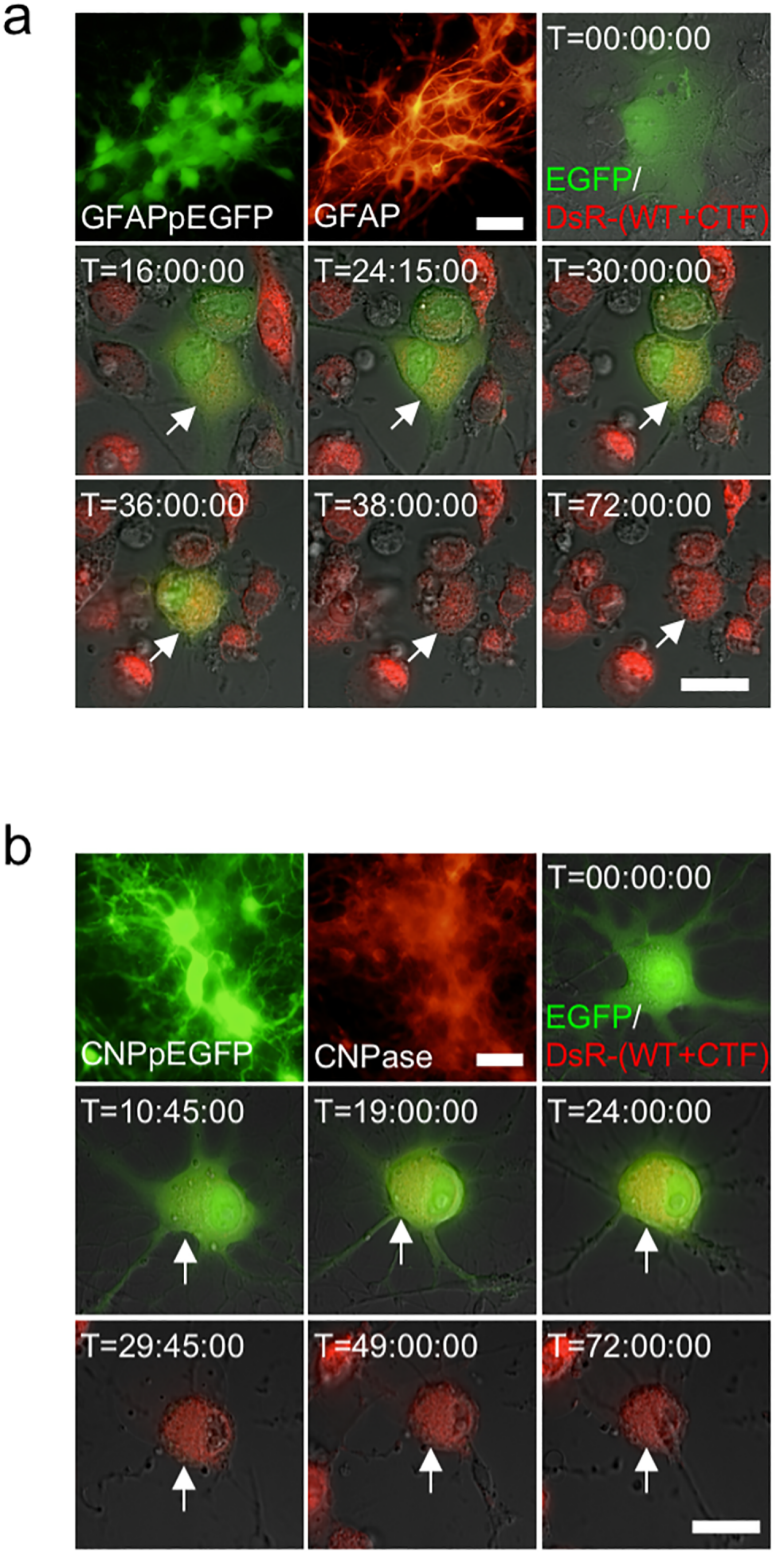
Adenovirus-transduced artificial TDP-43 aggregates formation in astrocyte and oligodendrocyte leads to cell death. Time-lapse imaging of adenovirus-transduced artificial TDP-43 cytoplasmic aggregate formation in glial cells. GFAP-positive 1464RGFAPpEGFP astrocytes (a) and CNP-positive 1464RCNPpEGFP oligodendrocytes (b) were transduced with AxDsR-WT.TDP43 and AxDsR-CTF.TDP43 (DsR-(WT+CTF)) (red) in the presence of MG-132. Transduced EGFP-positive astrocytes (green) (**a**) and oligodendrocytes (green) (**b**) are traced for 72 hrs, showing cytoplasmic aggregate formation, cell collapse and cell death (arrows). Scale bar = 20 μm.

As for adenovirus-transduced artificial TDP-43 cytoplasmic aggregate formation in astrocytes and oligodendrocytes, cell lines that express EGFP under the control of glial fibrillary acidic protein (GFAP) promoter (1464RGFAPpEGFP cells for astrocytes) and 2′,3′-cyclic nucleotide 3′-phosphodiesterase (CNP) promoter (1464RCNPpEGFP cells for oligodendrocytes) were established, respectively, and analyzed by time-lapse imaging. We transduced the differentiated EGFP-positive astrocytes and oligodendrocytes derived from 1464RGFAPpEGFP and 1464RCNPpEGFP cells, respectively, with adenoviruses expressing DsRed-tagged WT and CTF TDP-43 in the presence of MG-132. In a similar manner as observed in neuronal cells, cytoplasmic aggregates were formed in both astrocytes and oligodendrocytes, followed by cell death (Fig 7A and 7B).

### Spreading of TDP-43 aggregates

**Fig 8:**
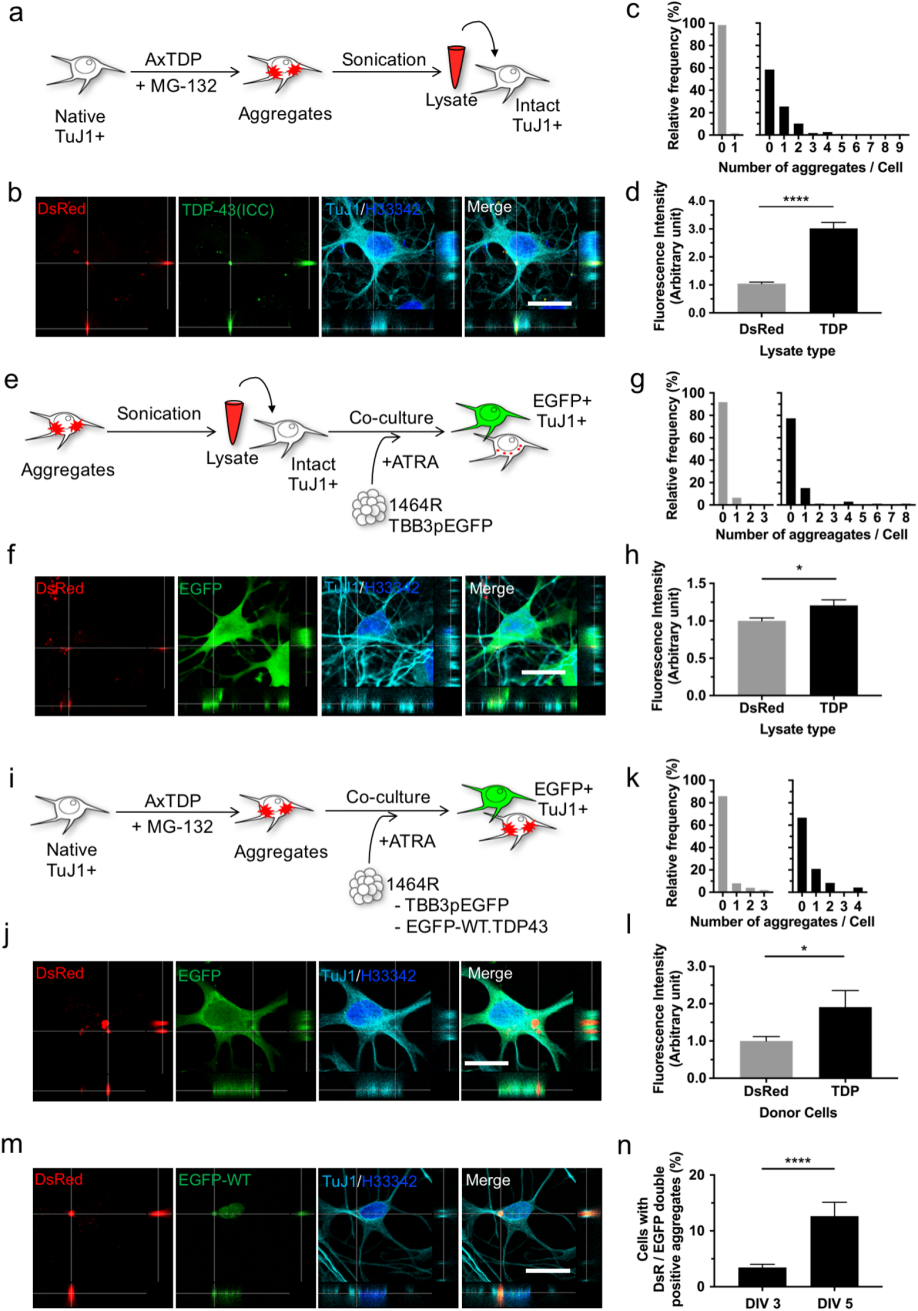
Cell-to-cell spreading of adenovirus-transduced artificial TDP-43 cytoplasmic aggregates. (**a, e, i**) Schematic drawings illustrating 3 different experiments. To prepare aggregate-harboring cells, 1464R neuronal cells were transduced with AxDsR-WT.TDP43 and AxDsR-CTF.TDP43 for 48 hrs and treated with 0.5 μM of MG-132 for an additional 24 hrs. Lysates from DsRed-transduced cells were used as control. **(c, g, k,n)** Aggregate-incorporated cells were represented with histogram and analyzed by Fisher’s exact test. **(d, h, l)** DsRed fluorescence intensity of each cell was measured and expressed as arbitrary unit. Calculated values were graphed with mean ± SEM and analyzed by Welch’s test. (**a-d**) Total cell lysates from aggregate-bearing cells were inoculated into new neurons (**a**). DsRed-positive TDP-43 aggregates are detected in the TuJ1-positive neurons **(b)**. Number of aggregates per cells in DsRed- (n = 163) and TDP-43- (n = 151) inoculated cells was graphed; ****p<0.0001 **(c)**. Relative DsRed fluorescence in each TuJ1-positive neurons was measured; ****p<0.0001 **(d)**. (**e-h**) New EGFP-expressing neurons were co-cultured with neurons that had been inoculated with cell lysates obtained from adenovirus-transduced /aggregate-bearing cells (**e**). DsRed-positive aggregates are detected in the cytoplasm of EGFP/TuJ1-positive neurons (**f**). Number of aggregates per cells co-cultured with DsRed (n = 170) and TDP (n = 172) transduced cells were graphed; ***p = 0.0002 **(g)**. Relative DsRed fluorescence in EGFP-positive neurons was calculated; *p = 0.0150 **(h)**. (**i-l**) Thoroughly washed TDP-43 aggregate-bearing cells were co-cultured with new EGFP-expressing neurons (**i**). DsRed-positive aggregates of ~2 μm in diameter are demonstrated in the cytoplasm of EGFP/TuJ1-positive neurons (**j**). Number of aggregates per cells co-cultured with DsRed (n = 50) or TDP (n = 48) transduced cells was graphed. *p = 0.0273 **(k)**. Relative DsRed fluorescence in EGFP-positive neurons was calculated. *p = 0.0476 **(l)**. (**m,n**) Thoroughly washed TDP-43 aggregates-bearing cells were co-cultured with EGFP-tagged WT TDP-43-expressing 1464R cells (1464REGFPWT.TDP-43) for 3 or 5 days (DIV 3 or DIV 5). (**m**) Transmitted aggregates (DsRed) (red) are co-localized with EGFP-WT (green). (**n**) The ratio of cells with DsRed- and EGFP-positive aggregates/cells expressing EGFP-WT.TDP43 and TuJ1 was calculated in DIV 3 and DIV 5. Each mean ratio was graphed. ****p<0.0001. After each experiment, cells were fixed and immunostained with TDP-43 (**a-d**) or GFP (**e-l**) (green) and TuJ1 antibodies (cyan) (**a-n**) and counterstained with Hoechst (H) 33342 (blue) (**a-n**). Scale bar = 20 μm. *p<0.05, ****p<0.0001.

Although cell-to-cell spreading of pathological aggregates is a plausible hypothesis in explaining the progressive nature of neurodegeneration observed in ALS and FTLD, there are no reports on time-lapse imaging of the direct transmission of TDP-43 aggregates. As shown in Fig 4D and S3 Movie, released TDP-43 aggregates from collapsed neurons were incorporated into neighboring neurons. Hence, we examined further the possibility of cell-to-cell spreading of adenovirus-transduced artificial TDP-43 cytoplasmic aggregates *in vitro*. To prepare the cells with TDP-43 aggregates, 1464R cells were transduced with DsRed-tagged TDP-43 WT and CTF followed by MG-132 treatment. Firstly, we sonicated the aggregate-bearing cells, obtained cell lysates, and inoculated new neurons with the lysates to examine the incorporation of aggregates into these cells (Fig 8A). Two days after inoculation, DsRed-positive granules immunostained for TDP-43 C-terminus were detected in TuJ1-positive neurons (Fig 8B). Quantitative analysis revealed that the number of DsRed aggregates (Fig 8C) and DsRed fluorescence intensity (Fig 8D) in TuJ1-positive cells were significantly higher as compared with cells inoculated with DsRed-alone lysates, indicating that TDP-43 aggregates were unambiguously incorporated into the new neurons. Secondly, to investigate whether incorporated TDP-43 could be transmitted to other cells, we co-cultured EGFP-expressing neurons with neurons that had been inoculated with cell lysates obtained from adenovirus-transduced, aggregate-bearing cells (Fig 8E). Two days after co-culture, DsRed-positive cytoplasmic small granules were detected in EGFP-expressing cells (Fig 8F). Quantitative analysis also showed that the number and intensity of DsRed aggregates were significantly increased (Fig 8G and Fig 8H). Thirdly, we thoroughly washed TDP-43 aggregate-bearing cells with medium to exclude remaining adenovirus and then co-cultured the cells with new EGFP-expressing neurons to investigate the cell-to-cell transmission of TDP-43 aggregates (Fig 8I). Two days after co-culture, fairly large TDP-43 aggregates of more than 2 μm in diameter were demonstrated in the cytoplasm of EGFP-expressing neurons (Fig 8J). Although DsRed-positive particles in EGFP-positive neurons were barely detectable when EGFP-expressing cells were co-cultured with cells expressing DsRed alone, the number of aggregates and DsRed fluorescence were significantly higher when co-cultured with TDP-43 aggregate-bearing cells. (Fig 8G and Fig 8L). This indicates that cell-to-cell spreading of TDP-43 aggregates occurs in this culture system. Finally, to investigate the possibilities of translocation of normal TDP-43 into the transmitted aggregates, TDP-43 aggregate-bearing cells were co-cultured with 1464R cells stably expressing EGFP-tagged WT TDP-43 (1464REGFPWT.TDP43). Three days later, we observed the EGFP-WT.TDP43 cells showing the co-localization of transmitted DsRed-positive aggregates with EGFP signals in the cytoplasm, indicating the seeding activity of the transmitted aggregates (Fig 8M). Then, the proportion of these cells were calculated; cells with DsRed- and EGFP-positive aggregates/cells expressing EGFP-WT.TDP43. Remarkably, the proportion of the double-positive aggregates was significantly increased at 5 days of co-culture (DIV 5) (12.64 ± 2.47%) compared with DIV 3 (3.44% ± 0.59%) (Fig 8N). Collectively, these results suggest that aggregated TDP-43 species are incorporated into neuronal cells, resistant to degradation within these cells, consecutively transmitted to adjacent neuronal cells and having seeding activities.

## Discussion

In our previous study, we showed that the formation of TDP-43 aggregates was enhanced by proteasome inhibition in adult rat and mouse motor neurons [26]. To investigate TDP-43 aggregate formation and cell death processes, we used neuronal and glial cells differentiated from a newly established neural stem cell line 1464R [26] and analyzed by the time lapse imaging methods. Many previous studies on TDP-43 aggregate formation have used neural cell lines such as SH-SY5Y, Neuro2a, and NSC34 [12,25,34,35] or non-neuronal cells [10,36]. However, significant differences in gene expression and epigenetic changes between such cell lines and primary cultured brain cells were reported [37]. The 1464R cell line used in the present study proliferates indefinitely without morphological alterations and, in the presence of retinoic acid, ceases dividing and differentiates into neuronal and glial cells that are very similar to primary cultured brain cells [38]. We believe that the 1464R cell line is a useful culture system that recapitulates *in vivo* neuronal and glial cells, since the TDP-43 aggregates that developed in differentiated 1464R cells were phosphorylated and ubiquitinated, and consisted of electron-dense granules, when the cells were treated with MG-132 following transduction with WT and CTF TDP-43. Furthermore, we demonstrated that phosphorylated and insoluble TDP-43 species observed in differentiated 1464R cells have biochemical properties similar to those seen in human ALS and FTLD brains by western blotting analyses following multiple fractionation of cell lysates. Many previous studies showed that cytoplasmic TDP-43 aggregates were formed by transfection of EGFP-tagged CTF TDP-43 and were regarded as recapitulation of TDP-43 pathology [10,34–36]. When transducing EGFP-tagged CTF TDP-43 in differentiated 1464R neuronal cells in the absence of MG-132, EGFP signals were expressed diffusely in the vast majority of cells. In these, 0.6% of the cells exhibited tiny punctated cytoplasmic aggregates that correspond to cytosolic inclusions of GFP-tagged CTF TDP-43 as described in the previous report [10]; these aggregates were small (~2 μm in diameter; not shown) as compared to massive cytoplasmic aggregates formed after MG-132 treatment as shown in the present study. This difference could be due to cell lines used and gene transduction methods (i.e., plasmid transfection vs. adenovirus-mediated transduction). Importantly, TDP-43 aggregates were more efficiently formed when WT and CTF TDP-43 were co-transduced in 1464R cells followed by MG-132 treatment. Our previous and present studies demonstrated that TDP-43 aggregates formation was enhanced by proteasome inhibition, which exhibited similar morphological properties as observed in ALS and FTLD-TDP pathology.

Although cytoplasmic aggregate formation is recognized as one of the pathological hallmarks of ALS and FTLD, toxicity of the aggregates and their involvement in cell death remains controversial [25,27,39]. The present time-lapse imaging analysis showed that, in the presence of MG-132, as cytoplasmic TDP-43 aggregates grew larger, cells were gradually swollen, eventually leading to cell death followed by collapse of the cytoplasmic membrane that was identified by dispersion of cytoplasmic EGFP fluorescence. The precise mechanisms of cell death triggered by TDP-43 and proteasome inhibition in our culture system should be a matter of the future research because several pathways were so far reported [40–43].

While detailed pathomechanisms of the neurodegenerative diseases remain largely unknown, the transmission of pathological aggregates is thought to be the principal causative event in disease progression [28–30,32]. Several reports have suggested that pathological aggregates containing disease-associated proteins are released and incorporated into healthy cells though various pathways, including those that involve exosomes [44,45] and tunnelling nanotubes [46]; incorporated aggregate seeds then convert the naive form of the disease-associated protein into the pathological form in affected cells [32,33]. This notion is recognized as prion-like propagation, and accumulating evidence favors this hypothesis for disease-associated proteins such as amyloid β, tau, α-synuclein, huntingtin, and SOD1 both *in vitro* and *in vivo* [29]. As for cell-to-cell propagation of TDP-43, Feiler et al. [47] reported that WT TDP-43 was transmitted via exosomes in a neuronal culture model. Nonaka et al. [48] described that the insoluble TDP-43 prepared from ALS and FTLD-TDP brains acted as seeds for TDP-43 aggregate formation in a self-templating manner. In the present study, we demonstrate that adenovirus-transduced artificial TDP-43 cytoplasmic aggregates are phosphorylated and ubiquitinated and are capable of acting as seeds for cell-to-cell spreading, which serve as a novel *in vitro* model of TDP-43 proteinopathy. In this context, proteasomal inhibition may also be a prerequisite for new aggregate formation in TDP-43-incorporated cells. However, it remains unclear whether the cell-to-cell transmission of aggregates in the presence of proteasome inhibitors is physiologically relevant and it cannot be clarified, in the present study, whether the cell death is directly caused by cell-to-cell spreading of TDP-43 aggregates and how proteasome inhibition itself affects neuronal viability. It could also be possible that uptake of particles via endocytosis is common in cell culture, but not necessarily dependent on TDP-43, especially CTF. Furthermore, another important question is how the transmitted aggregates were degraded in the recipient cells and affected by the proteasomal activity. These issues should be investigated in future studies.

In summary, we demonstrated cytoplasmic TDP-43 aggregate formation in neuronal and glial cells following adenoviral transduction of WT and CTF TDP-43 under MG-132 treatment. These TDP-43 aggregates were phosphorylated and ubiquitinated and consisted of electron-dense granules. Time-lapse imaging clearly demonstrated the time-course of TDP-43 aggregate formation, cell death, and spreading of aggregates. The relationship between formation and growth of cytoplasmic TDP-43 aggregates and cell death was evident as well as cell-to-cell spreading, where the released cytoplasmic aggregates were incorporated into adjacent neurons. These results recapitulate the TDP-43 pathology mainly observed in ALS and FTLD and suit the hypothesis of transmission of aggregated protein. Our model may provide new opportunities to reveal the pathomechanisms of TDP-43 proteinopathy and develop novel therapeutic strategies against ALS and FTLD-TDP.

## Methods

### Establishment of adult rat neural stem cell lines

Preparation of adult rat NSCs from 12-week old Fischer 344 rat brain stem tissue has been previously described [26]. All experiments were performed in accordance with Japanese national guidelines and regulations for scientific and ethical animal experimentation, and were approved by the Biosafety Committee (#14–038) and the Animal Care and Use Committee (#14022) of the Tokyo Metropolitan Institute of Medical Science. The NSCs were cultured in Neurobasal medium containing 2 mM L-glutamine, 2% B27 supplement (Thermo Fisher Scientific, Waltham, CA, USA), 10 ng/mL of fibroblast growth factor 2 (FGF2; Sigma, St. Louis, MO, USA), 10 ng/mL of epidermal growth factor (EGF; Sigma), 50 units/mL penicillin and 50 μg/mL streptomycin (Thermo Fisher) on 10-cm dishes coated with poly(2-hydroxyethylmethacrylate) (PHEMA; Sigma) to prevent cell attachment and were maintained in 5% CO_2_ at 37°C. Growing neurospheres after 3–4 weeks *in vitro* were mechanically dissociated and serially passaged in the same medium twice a week. One of the stable NSC lines, designated 1464R, was cultured further for more than 50 passages with no obvious morphological alterations. To establish rat neural stem cell lines stably expressing EGFP or Sirius [49] under the control of TBB3, GFAP, or CNP promoter, the CMV promoter regions of pEGFPN1 (Clontech, Palo Alto, CA USA) and Sirius/pcDNA (Addgene plasmid 51957) plasmids were replaced by rat TBB3 [50] (-490 to +34; AF458477), GFAP [51] (-1589 to -3), or CNP [52] (-1330 to -10) promoter fragment obtained by genomic PCR (pTBB3pEGFP, pTBB3pSirius, pGFAPpEGFP and pCNPpEGFP). The 1464R cells seeded on PHEMA-coated 3.5-cm dishes were transfected with these plasmids using Fugene 6 Transfection Reagent (Promega, Madison, WI, USA) according to the manufacturer’s instructions. After 2 days, the cells were expanded onto 10-cm dishes and maintained in the feeding medium containing 600 μg/mL G418 (Clontech). After 3–4 weeks, growing neurospheres were isolated, mechanically dissociated, and expanded in the same medium. The stably transfected cell lines, designated 1464RTBB3pEGFP, 1464RTBB3pSirius, 1464RGFAPpEGFP, and 1464RCNPpEGFP, were serially passaged in the feeding medium containing 200 μg/mL G418 twice a week. In order to use for TDP-43 seeding activity experiments, 1464R cells stably expressing EGFP-tagged human WT.TDP-43 (1464REGFPWT.TDP43) were established by the same transfection and culturing protocols as described above using EGFP-TDP-43 plasmid. To differentiate these 1464R cell lines into neuronal and glial cells, dissociated cells were seeded on poly-L-lysine (PLL)-coated 6-cm dishes or 9-mm ACLAR round coverslips (Allied Fibers & Plastics, Pottsville, PA, USA) at a density of 1–2 × 10^4^ cells per coverslip and maintained in F12 medium (Thermo Fisher) containing 5% fetal bovine serum (FBS), 0.5% N2 supplement (Thermo Fisher), 1% B27 supplement, 1 μM all-trans retinoic acid (ATRA; Sigma), 50 units/mL penicillin and 50 μg/mL streptomycin (Invitrogen) in 5% CO_2_ at 37°C for 2 days.

### *In vitro* adenovirus transduction

Preparation of recombinant adenovirus vectors encoding DsRed monomer (Clontech; #632466)-tagged human full length WT (AxDsR-WT.TDP43) and C-terminal fragment (CTF; 208-414aa of TDP-43) (AxDsR-CTF.TDP43) TDP-43 cDNAs, shRNAs for negative control (NC; GGAATCTCATTCGATGCATAC), and proteasome component PSMC1 (NM_057123; CGATGATAATCACGCCATTGT) coupled with EGFP (AxshNC/EGFP, AxshPSMC1/EGFP) have been previously described [26]. Additionally, in the present study, adenovirus vectors expressing EGFP-tagged human WT and CTF TDP-43 (AxEGFP-WT.TDP43, AxEGFP-CTF.TDP43) were prepared by the same procedure as described [26]. Neurons and glial cells differentiated from 1464R NSCs on PLL-coated 6-cm dishes or 9-mm coverslips were transduced with the TDP-43 adenoviruses at a multiplicity of infection of 50. Forty-eight hrs later, the cells were refed with the media with or without 0.5 μM MG-132 (Sigma) and further incubated for 24 hrs. In some experiments, the cells were simultaneously transduced with AxDsR-WT.TDP43, AxDsR-CTF.TDP43 and AxshPSMC1/EGFP for 72 hrs instead of MG-132 treatment. The treated cells on the dishes or coverslips were lysed for western blotting or fixed for immunocytochemistry and electron microscopy, respectively, as described below. DsRed, EGFP, or Sirius fluorescence was examined under an IX70 inverted fluorescence microscope equipped with DP72 CCD camera (Olympus, Tokyo, Japan) prior to cell lysate preparation or fixation.

### Cell fractionation

For sarkosyl fractionation, cells were lysed and sonicated in Tris buffer (50 mM Tris-HCl pH 7.5, 150 mM NaCl, 5 mM EDTA and 5 mM EGTA) containing phosphatase inhibitors (5 mM NaF and 2 mM Na_3_VO_4_) and 1× protease inhibitor cocktail (Millipore, Billerica, MA, USA), followed by centrifugation at 160,000 × *g* for 20 min at 4°C. The supernatants were collected as Tris soluble fraction (termed T). The pellets were lysed and sonicated in 1% TritonX-100 in Tris buffer, followed by centrifugation at 160,000 × *g* for 20 min at 4°C and the supernatants were collected as Triton X-100 soluble fraction (termed X). The pellets were lysed and sonicated in 1% (w/v) sarkosyl in Tris buffer and incubated for 30 min at 37°C. The lysates were centrifuged at 160,000 × *g* for 20 min at 4°C, and the supernatants were collected as sarkosyl soluble fraction (termed S). The insoluble pellets (termed P) were lysed and sonicated in 1x SDS-sample buffer containing 2-mercaptoethanol (ME).

For RIPA/urea fractionation, cells were lysed in RIPA buffer [50 mM Tris-HCl pH 8.0, 150 mM NaCl, 5 mM EDTA, 1% Nonidet P-40, 0.5% sodium deoxycholate and 0.1% sodium dodecyl sulfate (SDS)] containing phosphatase inhibitors as above, sonicated and incubated for 30 min on ice followed by centrifugation at 100,000 × *g* for 30 min at 4°C. The supernatants were collected and kept on ice as RIPA soluble fraction (termed R), and the pellets were lysed and sonicated in urea buffer [30 mM Tris-HCl, pH 8.5, 7 M urea, 2 M thiourea and 4% 3-(3-cholamidepropyl) dimethylammonio-1-propanesulphonate (CHAPS)] and centrifuged at 100,000 × *g* for 30 min at 20°C. The supernatants were collected as urea soluble fraction (termed U). Protein concentration of each fraction was determined with BCA Protein Assay kit (Pierce, Rockford, IL, USA).

### Western blot analysis

Ten—fifteen micrograms of cell lysate were electrophoresed on 10% SDS/polyacrylamide gels under reduced conditions and transferred to PVDF membrane (ATTO, Tokyo, Japan). The blotted membrane was blocked with 5% skim milk and incubated overnight with rabbit anti-TDP-43 C-terminus (405–414) or phospho-TDP-43 (pSer409/S410) antibodies (Cosmo Bio Co., LTD., Tokyo, Japan) at dilutions of 1:3,000, followed by incubation with HRP-conjugated anti-rabbit IgG (1:5,000; GE Healthcare, Buckinghamshire, UK). Reactions were visualized by enhanced chemiluminescence detection using an ECL Western blotting detection kit (GE Healthcare) and the images were captured and modified with Ez-Capture (ATTO).

### Immunocytochemistry

Cells were fixed with 4% paraformaldehyde in phosphate buffered saline (PBS), permeabilized with 100% methanol, washed with PBS, and immunostained overnight at 4°C with the following primary antibodies at 1:200 dilutions; mouse monoclonal TuJ1 (R&D systems, Minneapolis, MN, USA), rabbit anti-phosphoTDP-43 (pSer409/410) (Cosmo Bio), rabbit anti-TDP-43 C-terminus (Abcam, Cambridge, MA, USA), rabbit anti-GFP (Abcam), rabbit anti-GFAP (DAKO, Glostrup, Denmark), rabbit anti-ubiquitin (DAKO) and rabbit anti-CNP (Cell Signaling Technology, Danvers, MA, USA). The cells were then incubated with Alexa Fluor 488- or 633-conjugated goat anti-rabbit or anti-mouse antibodies (Thermo Fisher) at 1:400 dilutions for 45 min at room temperature, followed by incubation for 15 min with 2 μg/mL Hoechst 33342 (Thermo Fisher). After washings, coverslips were mounted on glass slides with Gelvatol (20% glycerol/10% polyvinyl alcohol in 0.1 M Tris-HCl pH 8.0). Immunostained cells were examined under an AX80TR microscope equipped with a DP70 CCD camera (Olympus) or a TCS SP8 confocal microscope (Leica, Wetzlar, Hessen, Germany).

### Electron microscopy

For electron microscopic analysis, cells on coverslips were fixed with 3% glutaraldehyde in 0.1 M PBS. After washings, cells were post-fixed with 1% osmium tetroxide in 0.1 M phosphate buffer, pH 7.4, dehydrated through graded ethanol steps, and embedded in Epon 812. The coverslips were peeled off and ultrathin sections were horizontally made, stained with uranyl acetate and lead citrate, and examined under a Hitachi H-7650 electron microscope.

### Time-lapse imaging

Time-lapse live cell fluorescence imaging was performed using a DeltaVision microscopy system (GE, Fairfield, CT, USA). The 1464RTBB3pEGFP or 1464RTBB3pSirius cells were seeded and differentiated on a PLL-coated 35-mm glass bottom dish (Greiner, Pleidelsheim, Germany) for 2 days and transduced with AxDsR-WT.TDP43, AxEGFP-WT.TDP43, AxDsR-CTF.TDP43, and/or AxEGFP-CTF.TDP43 for another 2 days. Two hours before imaging, incubation chambers were pre-warmed to 37°C and filled with 5% CO_2_ to start running the program. Prior to time-lapse imaging, cells were fed with new medium with or without 0.5 μM MG-132. In some experiments, cells were co-transduced with AxshNC/EGFP or AxshPSMC1/EGFP in place of MG-132 treatment. The dish was immediately set on a microscopic stage and the object areas were determined with the aid of EGFP (excitation at 475/28 nm and emission at 525/48 nm) and DsRed (excitation at 542/27 nm and emission at 597/45 nm) fluorescence observation. Sirius, one of the blue fluorescent proteins that have maximum excitation at 355 nm and maximum emission at 425 nm with improved photostability [49], was captured (excitation at 390/18 nm and emission at 435/48 nm) only once just before running the time-lapse experiment to avoid cell damages by ultraviolet light. The time-lapse program was successfully performed at 15 or 20 min intervals for 72 hrs, resulting in 289 images per region of interest per channel. Dead cells were determined when tracing cells were atrophied or EGFP fluorescence were disappeared. EGFP positive cells were counted at 24 and 48 hrs time points. Red auto-fluorescence due to cell death that also showed another fluorescence such as green and far-red were excluded from the analysis. Acquired images were deconvolved and converted into movies as “.mp4” format by SoftWoRx software (GE).

### TDP-43 spreading experiments

To examine spreading of naked TDP-43 aggregates to naive cells, neuronal and glial cells differentiated from 1464R NSCs were cultured on PLL-coated 6-well plates (4 × 10^5^ cells/well), transduced with AxDsR-WT.TDP43 and AxDsR-CTF.TDP43, and subsequently treated with MG-132. The cells were rinsed twice with pre-chilled PBS and harvested by cell scraper. Collected cells were sonicated and the cell lysates corresponding to 2 × 10^4^ transduced cells were inoculated onto 2 × 10^4^ non-treated differentiated cells derived from 1464R cells on coverslips. Cells were fixed after 48 hrs and processed for immunocytochemistry as described above.

To examine cell-to-cell spreading of TDP-43 aggregates, differentiated 1464R cells that had been inoculated with sonicated cell lysates containing TDP-43 aggregates as described above were washed and seeded on naive differentiated 1464RTBB3pEGFP cells on coverslips. In some experiments, differentiated 1464R cells were treated with adenoviruses and MG-132, thoroughly washed in medium, and seeded on naive differentiated 1464RTBB3pEGFP cells (1 × 10^4^) on coverslips. Cells were fixed after 48 hrs for immunocytochemistry.

To examine the seeding activity of the aggregates, differentiated 1464R cells (3 × 10^3^) were treated with adenoviruses and MG-132, thoroughly washed in medium, dissociated, and overlayed on the differentiated 1464REGFPWT.TDP43 cells (3 × 10^4^) maintained on coverslips. Cells were fixed after 3 or 5 days for immunocytochemistry. DsRed fluorescence and maximum fluorescence in TuJ1- or EGFP-positive area were quantified with ImageJ software (version 1.49, NIH, Bethesda, MD, USA).

### Statistics

For cell survival analysis, Kaplan-Meier survival curve was drawn from time-lapse data and the statistical significance was analyzed by Log-Rank test. The number of EGFP-positive cells with different time points was analyzed with two-way ANOVA with Sidak’s multiple comparisons test. For spreading experiment, aggregates positive cells were analyzed by Mann-Whitney’s test and relative intensity of fluorescence were analyzed by Welch’s test. The proportion of the cells with DsRed-positive aggregates was analyzed by Fisher’s exact test. All bar graphs showed mean ± standard error of the mean (SEM). Measured results were analyzed with two-tailed test and significance level was set at p<0.05. All results were analyzed and graphed with Prism 7 software (GraphPad Software, San Diego, CA, USA).

## Supporting information

S1 Fig Combined DsRed-tagged WT and CTF TDP-43 transduction in the presence of MG-132 induces cytoplasmic aggregates in 1464R cells. (**a-d**) Differentiated neurons were transduced with AxDsR-WT.TDP43 (DsR-WT) (**a,c**) or AxDsR-CTF.TDP43 (DsR-CTF) (**b,d**) (red) followed by the treatment with DMSO (**a,b**) or 0.5 μM MG-132 (c,d). Fixed cells were immunostained with phosho-TDP-43 (pS409/S410) (green) and TuJ1 (white), and counterstained with Hoechst 33342 (blue). Arrows indicate cytoplasmic aggregates. Scale bar = 20μm.
(TIF)

S2 Fig EGFP-tagged WT and CTF TDP-43 expression shows similar properties to DsRed-tagged TDP-43 expression. (**a-f**) Differentiated neurons were transduced with AxEGFP-WT.TDP43 (EGFP-WT) (a,d), AxEGFP-CTF.TDP43 (EGFP-CTF) (**b,e**) or both (c,f) (green) followed by the treatment with DMSO (**a-c**) or 0.5 μM MG-132 (**d-f**). Fixed cells were immunostained with phosho-TDP-43 (pS409/S410) (green) and TuJ1 (white) and counterstained with Hoechst 33342 (blue). Arrows indicate cytoplasmic aggregates. Scale bar = 20μm.
(TIF)

S3 Fig Proteasome inhibitiors accelerate the formation of TDP-43 aggregates. (**a-c**) Differentiated neurons were transduced with AxDsR-WT.TDP43 and AxDsR-CTF.TDP43 (DsR-(WT+CTF)) (red) followed by the treatment with DMSO (**a**), 0.5 μM MG-132 (**b**), or 1 μM lactacystin (**c**) for 24 hrs. Fixed cells were immunostained with phosho-TDP-43 (pS409/S410) (green) and TuJ1 (white) and counterstained with Hoechst 33342 (blue). Arrows indicate cytoplasmic aggregates. Scale bar = 20μm. (**d-e**) Differentiated neurons transduced with AxDsR-WT.TDP43 and AxDsR-CTF.TDP43 (DsR-(WT+CTF)) were treated with DMSO, 0.5 μM MG-132, 1 μM lactacystin, 0.1 μM epoxomicin, or 26 μM ALLN for 24 hrs. Sarkosyl soluble (S) and Sarkosyl insoluble (P) fractions were immunoblotted with antibodies for phosho (p)-TDP-43 (pS409/S410) (**d**) or TDP-43 (405–410) (**e**). The 72 and 50 kDa bands correspond to non-phosphorylated DsRed-tagged WT and CTF TDP-43, respectively. TDP-43 antibody also detects endogenous rat TDP-43 (arrowhead).
(TIF)

S4 Fig Insoluble cytoplasmic aggregates are formed by EGFP-tagged TDP-43 adenoviruses in the presence of MG-132. (**a**) Time-lapse imaging of AxEGFP-WT.TDP43 and AxEGFP-CTF.TDP43 (EGFP-(WT+CTF); gray scale in top panel and green in bottom panels, respectively)-transduced 1464RTBB3pSirius neurons in the presence of MG-132. Cytoplasmic TDP-43 aggregates (arrow) are formed and remained in the insoluble material after cell collapse. See also S5 Movie. (**b**) Time-lapse imaging of AxDsR-WT.TDP43 (DsR-WT; gray scale in top panel and red in bottom panel, respectively) and AxEGFP-CTF.TDP43 (CTF; gray scale in middle panel and green in bottom panel, respectively)-transduced with 1464RTBB3pSirius neurons in the presence of MG-132. Cytoplasmic TDP-43 aggregates shows both EGFP and DsRed fluorescence (arrow). Scale bar = 20 μm. See also S6 Movie.
(TIF)

S1 Movie Time-lapse video of 1464RTBB3pEGFP-derived neuronal cells (green) transduced with AxDsR-WT.TDP43 and AxDsR-CTF.TDP43 (red) (for Fig 4B). Images were captured by every 15 min. The obtained serial images were converted into movie with 5 frames/second. Scale bar = 15 μm.
(MP4)

S2 Movie Time-lapse video of 1464RTBB3pEGFP-derived neuronal cells (green) transduced with AxDsR-WT.TDP43 and AxDsR-CTF.TDP43 (red) followed by 0.5 μM MG-132 (for Fig 4C). Images were captured by every 15 min. The obtained serial images were converted into movie with 5 frames/second. Scale bar = 15 μm.
(MP4)

S3 Movie Time-lapse imaging of 1464RTBB3pEGFP-derived neuronal cells (green) transduced with AxDsR-WT.TDP43 and AxDsR-CTF.TDP43 (red) followed by 0.5 μM MG-132 (for Fig 4D). Images were captured by every 20 min. The obtained serial images were converted into movie with 5 frames/second. Scale bar = 15 μm.
(MP4)

S4 Movie Time-lapse imaging of 1464RTBB3pSirius-derived neuronal cells transduced with AxEGFP-WT.TDP43 (green) and AxDsR-CTF.TDP43 (red) followed by 0.5 μM MG-132 (for Fig 5A). Images were captured by every 15 min. The obtained serial images were converted into movie with 5 frames/second. Scale bar = 15 μm.
(MP4)

S5 Movie Time-lapse imaging of 1464RTBB3pSirius-derived neuronal cells transduced with AxEGFP-WT.TDP43 and AxEGFP-CTF.TDP43 (green) followed by 0.5 μM MG-132 (for Fig A in S4 Fig). Images were captured by every 15 min. The obtained serial images were converted into movie with 5 frames/second. Scale bar = 15 μm.
(MP4)

S6 Movie Time-lapse imaging of 1464RTBB3pSirius-derived neuronal cells transduced with AxDsR-WT.TDP43 (red) and AxEGFP-CTF.TDP43 (green) followed by 0.5 μM MG-132 (for Fig B in S4 Fig). Images were captured by every 15 min. The obtained serial images were converted into movie with 5 frames/second. Scale bar = 15 μm.
(MP4)
