# Supplementary figures and images for: Formation and spreading of TDP-43 aggregates in cultured neuronal and glial cells demonstrated by time-lapse imaging
Source: PLoS One. 2017 Jun 9;12(6):e0179375. doi: 10.1371/journal.pone.0179375 (PMC5466347; doi:10.1371/journal.pone.0179375)

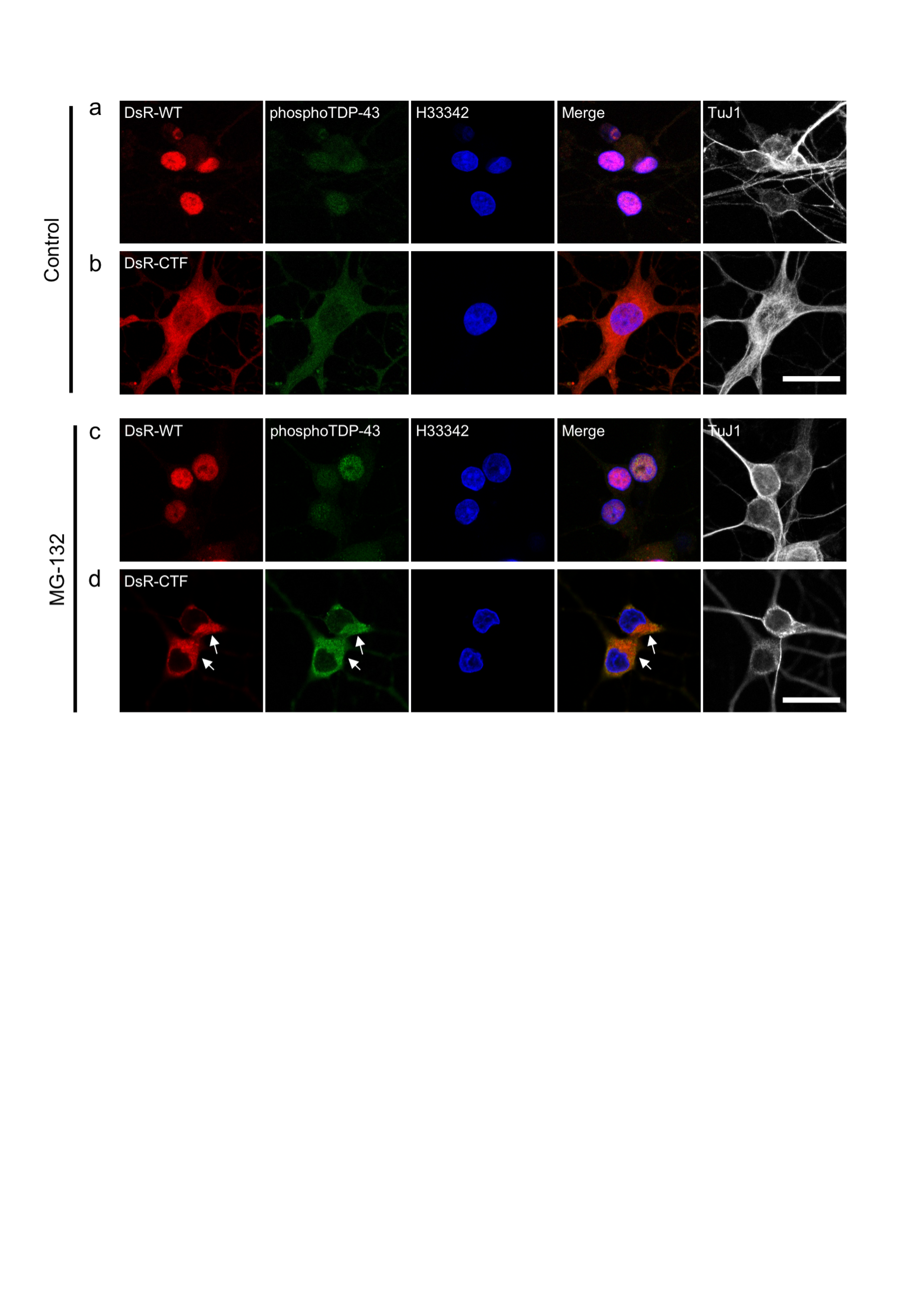

Supplement: S1 Fig — (a-d) Differentiated neurons were transduced with AxDsR-WT.TDP43 (DsR-WT) (a,c) or AxDsR-CTF.TDP43 (DsR-CTF) (b,d) (red) followed by the treatment with DMSO (a,b) or 0.5 μM MG-132 (c,d). Fixed cells were immunostained with phosho-TDP-43 (pS409/S410) (green) and TuJ1 (white), and counterstained with Hoechst 33342 (blue). Arrows indicate cytoplasmic aggregates. Scale bar = 20μm. (TIF) [file pone.0179375.s001.tif]

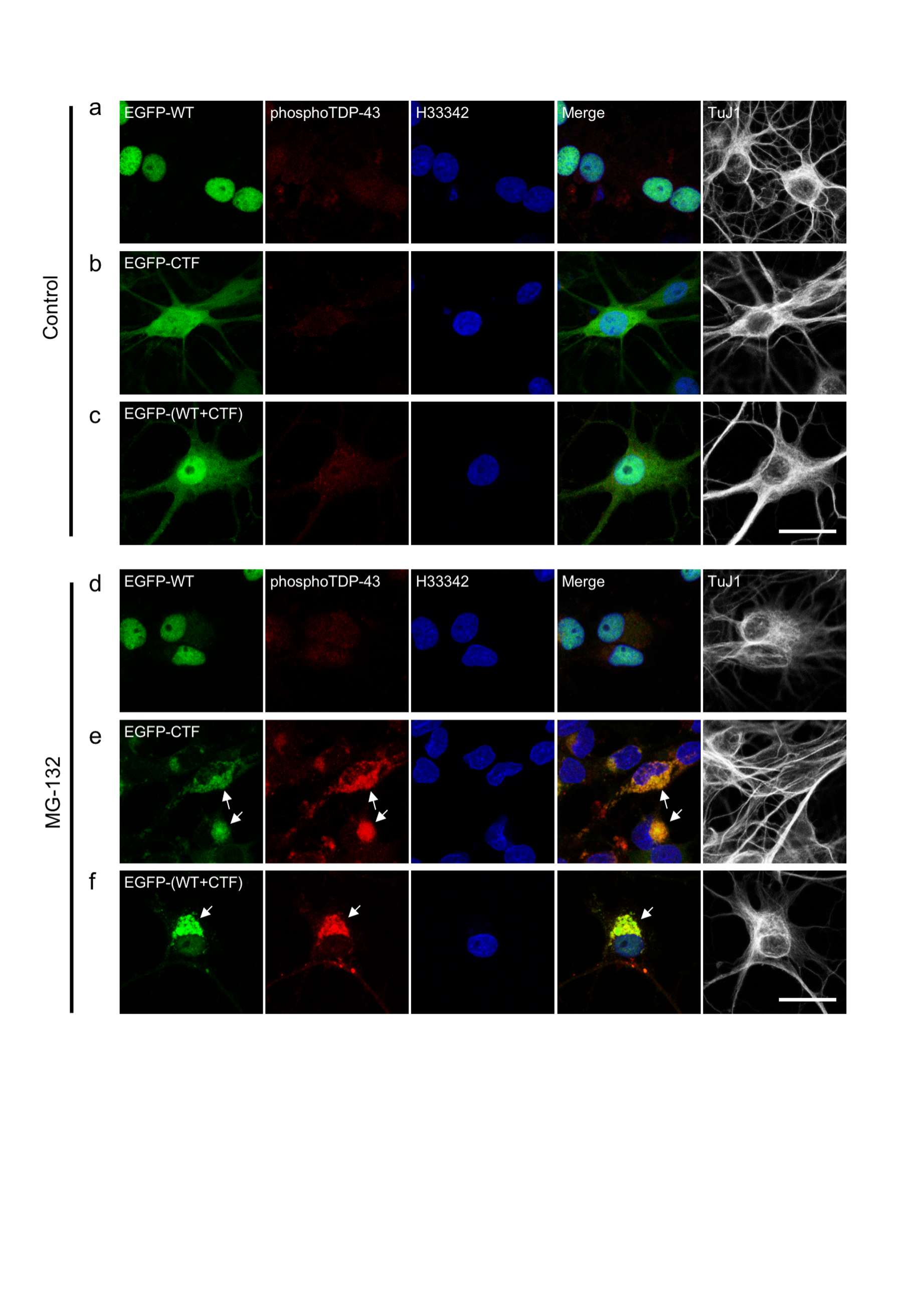

Supplement: S2 Fig — (a-f) Differentiated neurons were transduced with AxEGFP-WT.TDP43 (EGFP-WT) (a,d), AxEGFP-CTF.TDP43 (EGFP-CTF) (b,e) or both (c,f) (green) followed by the treatment with DMSO (a-c) or 0.5 μM MG-132 (d-f). Fixed cells were immunostained with phosho-TDP-43 (pS409/S410) (green) and TuJ1 (white) and counterstained with Hoechst 33342 (blue). Arrows indicate cytoplasmic aggregates. Scale bar = 20μm. (TIF) [file pone.0179375.s002.tif]

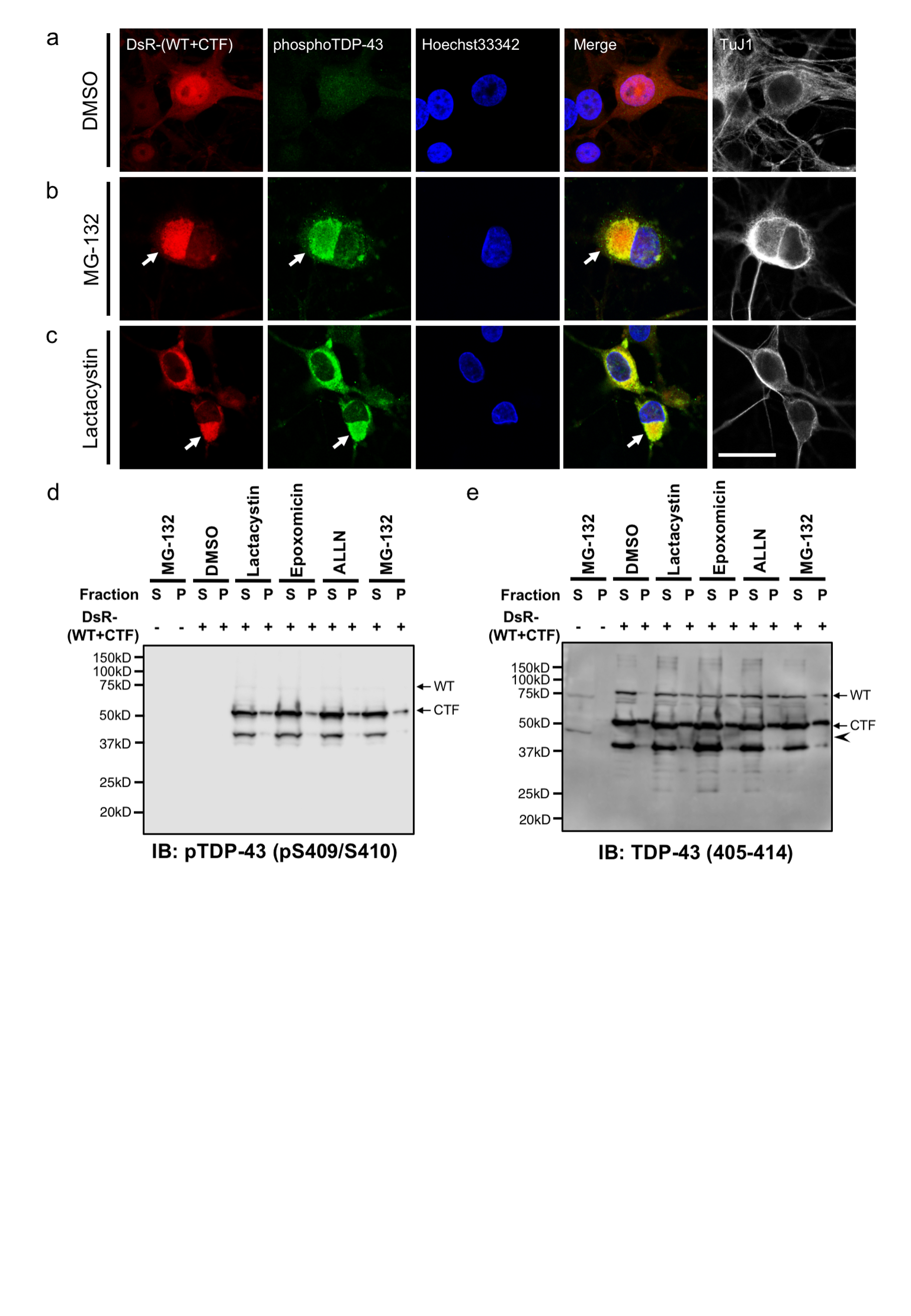

Supplement: S3 Fig — (a-c) Differentiated neurons were transduced with AxDsR-WT.TDP43 and AxDsR-CTF.TDP43 (DsR-(WT+CTF)) (red) followed by the treatment with DMSO (a), 0.5 μM MG-132 (b), or 1 μM lactacystin (c) for 24 hrs. Fixed cells were immunostained with phosho-TDP-43 (pS409/S410) (green) and TuJ1 (white) and counterstained with Hoechst 33342 (blue). Arrows indicate cytoplasmic aggregates. Scale bar = 20μm. (d-e) Differentiated neurons transduced with AxDsR-WT.TDP43 and AxDsR-CTF.TDP43 (DsR-(WT+CTF)) were treated with DMSO, 0.5 μM MG-132, 1 μM lactacystin, 0.1 μM epoxomicin, or 26 μM ALLN for 24 hrs. Sarkosyl soluble (S) and Sarkosyl insoluble (P) fractions were immunoblotted with antibodies for phosho (p)-TDP-43 (pS409/S410) (d) or TDP-43 (405–410) (e). The 72 and 50 kDa bands correspond to non-phosphorylated DsRed-tagged WT and CTF TDP-43, respectively. TDP-43 antibody also detects endogenous rat TDP-43 (arrowhead). (TIF) [file pone.0179375.s003.tif]

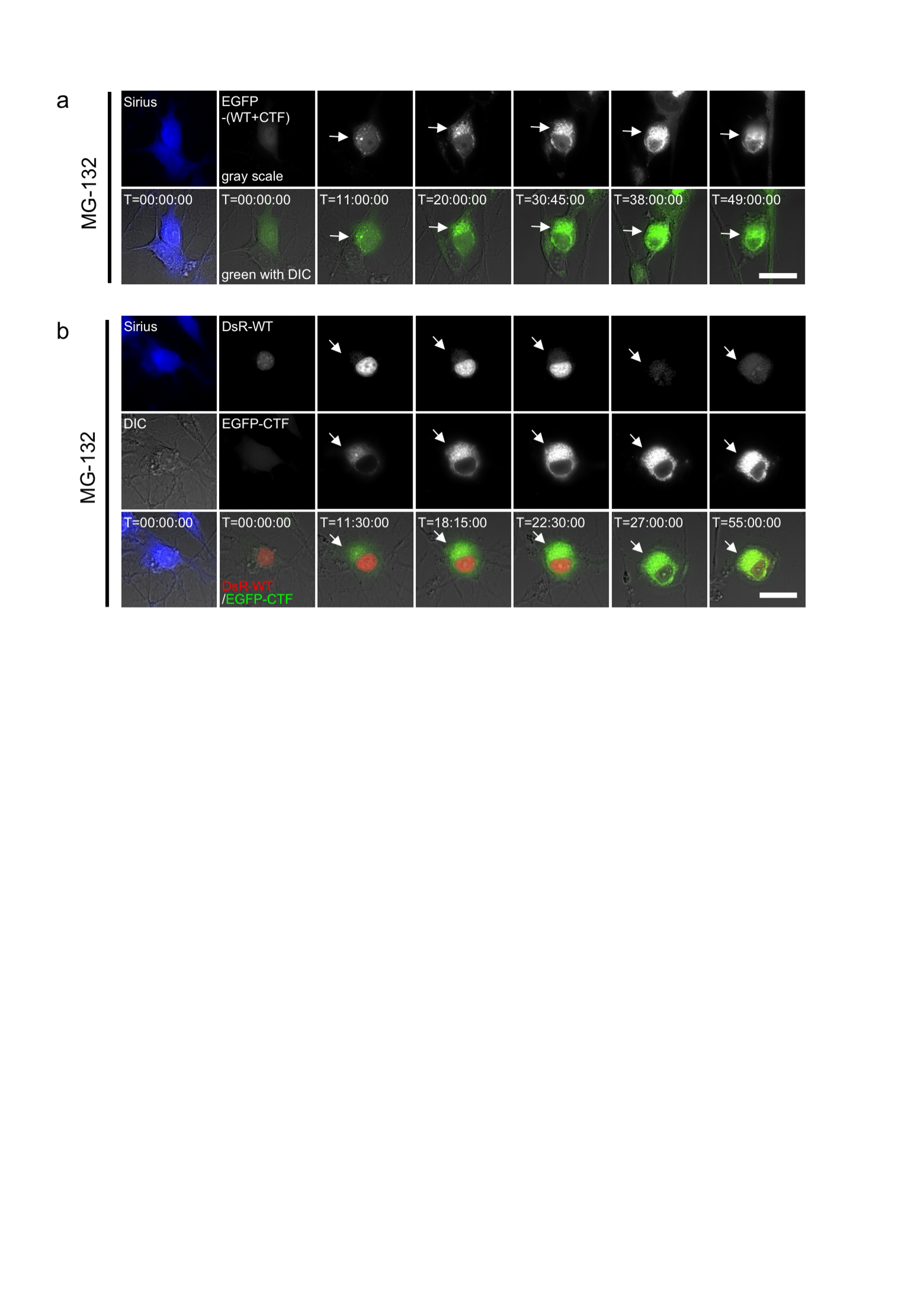

Supplement: S4 Fig — (a) Time-lapse imaging of AxEGFP-WT.TDP43 and AxEGFP-CTF.TDP43 (EGFP-(WT+CTF); gray scale in top panel and green in bottom panels, respectively)-transduced 1464RTBB3pSirius neurons in the presence of MG-132. Cytoplasmic TDP-43 aggregates (arrow) are formed and remained in the insoluble material after cell collapse. See also S5 Movie. (b) Time-lapse imaging of AxDsR-WT.TDP43 (DsR-WT; gray scale in top panel and red in bottom panel, respectively) and AxEGFP-CTF.TDP43 (CTF; gray scale in middle panel and green in bottom panel, respectively)-transduced with 1464RTBB3pSirius neurons in the presence of MG-132. Cytoplasmic TDP-43 aggregates shows both EGFP and DsRed fluorescence (arrow). Scale bar = 20 μm. See also S6 Movie. (TIF) [file pone.0179375.s004.tif]
